# The Long-Term Esthetic and Radiographic Outcome of Implants Placed in the Anterior Maxilla after Ridge Preservation, Combining Bovine Xenograft with Collagen Matrix

**DOI:** 10.3390/dj12030080

**Published:** 2024-03-20

**Authors:** Leonidas Batas, Vithleem Xanthopoulou, Maria Gnigou, Triantafyllia Vagdouti, Ioannis Fragkioudakis, Ioannis Vouros

**Affiliations:** Department of Preventive Dentistry, Periodontology and Implant Biology, School of Dentistry, Aristotle University of Thessaloniki, 541 24 Thessaloniki, Greece; lbatas76@gmail.com (L.B.); thelmaxan@yahoo.gr (V.X.); dent.gnigou@gmail.com (M.G.); phillievagdouti@hotmail.com (T.V.); ifragkio@gmail.com (I.F.)

**Keywords:** implants, esthetics, regeneration, collagen, PES

## Abstract

The objective of the study was to evaluate the long-term esthetic and radiographic results of implants placed in the anterior maxilla after ridge preservation, combining bovine xenograft with collagen matrix. Fifteen patients who required a single tooth extraction because of fracture, root resorption, or extended caries were included in the study. After extraction, all sites were grafted using Deproteinized Bovine Bone Mineral (DBBM) with collagen and covered by a resorbable collagen matrix (CM). Five months after socket grafting, implants were successfully installed. The implant diameter range was between 3.8 and 4.2 mm. All patients were monitored for over 7 years, both clinically and radiographically. Three independent observers evaluated the long-term esthetic outcome, employing the Pink Esthetic Score (PES) technique. Over a period exceeding seven years, a 100% survival rate was observed for all 15 implants, with minimal marginal bone loss. The mean PES was 11.40 (±1.44) at the first assessment and 11.38 (±1.63) at the second assessment. The difference was not statistically significant (*p* = 0.978), and the scores of PES measurements indicated excellent esthetic results even after seven years. Based on these preliminary results, it seems that placing collagen bovine bone in a fresh extraction socket, covered with a collagen matrix, can preserve the alveolar ridge and provide long-term stable esthetic results.

## 1. Introduction

Following tooth extraction, a sequence of events arises regarding modeling and remodeling processes during socket healing [1]. This chronic irreversible process leads to qualitative and quantitative changes at the edentulous site and an almost 50% reduction of the alveolar ridge within the first six months [1,2,3]. Such changes to the alveolar ridge may reduce the volumetric soft tissue thickness and keratinized mucosa. This leads to esthetic problems around the fixed dental prosthesis or renders the installation of dental implants more challenging, requiring guided bone regeneration [3,4,5,6]. It has also been established that even immediate implant placement, in a fresh extraction socket, fails to prevent bone resorption, especially of the buccal wall, often resulting in esthetic deterioration [4,7,8,9,10].

Over the past two decades, multiple studies have been conducted to test the efficacy of alveolar ridge preservation (ARP). These have included minimally traumatic tooth extraction followed by immediate grafting of the extraction sockets using particulate bone grafts or substitutes and guided bone regeneration (GBR), with or without bone grafts or substitutes. A variety of bone grafting materials have been used for socket grafting (ARP-SG) including allografts, xenografts, alloplasts, autogenous bone, and other growth factors. Most systematic reviews agree that ARP-SG reduces alveolar bone resorption in horizontal and vertical dimensions [11,12,13,14].

The biocompatibility of Deproteinized Bovine Bone Mineral (DBBM) in the extraction sockets and its possible integration into the newly formed bone have been evaluated in quite a number of preclinical and clinical studies [15,16,17].

These studies were performed to determine the composition of tissues formed six months after the placement of DBBM into the extraction socket. They all demonstrated delayed healing but successful preservation of the alveolar ridge with reduced dimensional loss when compared with ungrafted sites [18]. It has also been established that there is clinically sufficient quality and quantity of bone, allowing for appropriate implant placement after nine months of healing [18].

In recent years, advancements in bone grafting materials have led to the development of formulations combining deproteinized bovine bone mineral (DBBM) granules with porcine collagen. This combination, consisting of 90% DBBM and 10% porcine collagen, has shown improved formability and handling characteristics compared to traditional grafting materials [17]. Notably, studies have demonstrated successful preservation of alveolar ridge dimensions when such grafting materials are placed alone in fresh extraction sockets, with retrospective analyses indicating stable implants and minimal bone loss even up to 10 years post-surgery [19].

In addition to bone grafting materials, the management of soft tissues around extraction sockets is critical for achieving optimal esthetic outcomes in implant-supported restorations. Historically, techniques involving palatal soft tissue grafts presented drawbacks such as increased operative time, risk of graft necrosis, and patient morbidity due to tissue harvesting from the palate. However, recent innovations have introduced xenogeneic, porcine non-cross-linked bilayer resorbable collagen matrices (CMs) designed to promote soft tissue regeneration, minimize pain, and reduce complications [19,20].

These CMs, comprising pure type I and III collagen, offer a ready-to-use solution for sealing extraction sockets, particularly in ridge preservation procedures with preserved buccal walls [21]. The compact layer of collagen facing the oral cavity promotes tissue adherence and marginal adaptation, facilitating favorable wound healing, while the porous, spongy structure of the second layer encourages tissue integration and early vascularization [22,23]. Clinical and histological studies have demonstrated successful revascularization, re-epithelialization, and integration of CMs into surrounding tissues without inflammation, resulting in improved soft tissue thickness, width, and color match compared to spontaneous healing [20,21].

Despite advancements in socket preservation techniques, significant heterogeneity and a lack of consensus remain regarding outcomes such as the need for further augmentation, esthetic factors, and implant failure [24]. Furthermore, most studies report short-term clinical results, typically less than 36 months [25].

To evaluate soft tissue esthetic outcomes, techniques such as the Pink Esthetic Score (PES) have been proposed. PES provides an objective assessment of soft tissue esthetics in implant-supported restorations and various surgical or prosthodontic treatment protocols [26], with demonstrated reproducibility in past studies [27,28].

Given the aforementioned advancements and considerations, the primary objective of the present study is to describe a technique for grafting fresh extraction sockets using DBBM bone grafting materials supplemented with collagen (Geistlich Bio-Oss^®^ Collagen, Geistlich, Wolhusen, Switzerland) and covered with a collagen matrix (Mucograft^®^ Seal, Geistlich, Wolhusen, Switzerland). The study aims to evaluate the long-term stability of soft tissue esthetic outcomes and radiographic marginal bone loss in implants placed at these sites, with a follow-up period exceeding seven years.

The study’s secondary objective is to evaluate the reproducibility of the Pink Esthetic Score technique (PES). 

## 2. Methods

### 2.1. Study Sample

Patients participating in this retrospective case series study required single tooth extraction in the esthetic zone of the maxilla due to fracture, root resorption, or subgingival caries (Table 1). No molars were included. All patients were periodontally healthy and free of contraindications relating to this procedure, such as uncontrolled diabetes, long-term steroid usage, heavy smoking, or blood disorders. 

Patients were offered alternative treatment plans and selected ones requiring tooth extraction, socket preservation, and implant placement. Patients were informed about the nature of the study and asked to sign an informed consent form.

### 2.2. Surgical Procedures—Implant Rehabilitation

**Figure 1:**
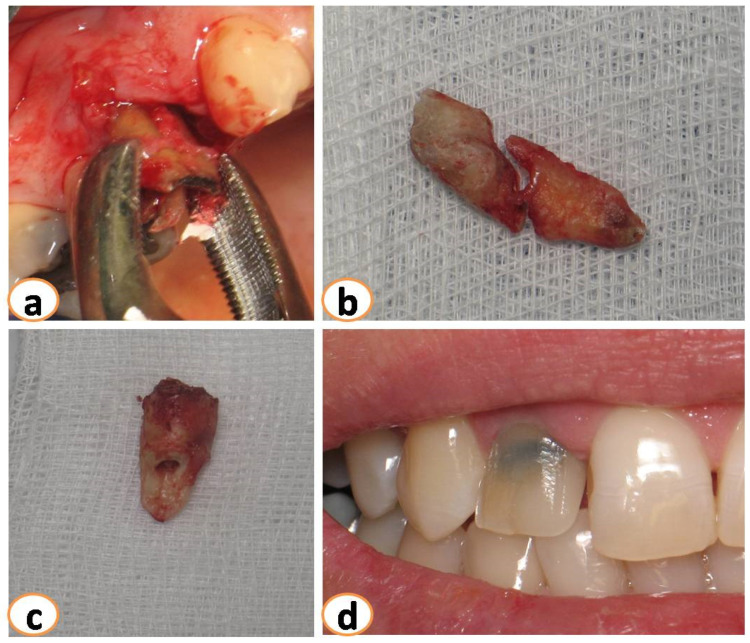
(**a**): Minimal traumatic extractions were performed. Teeth extracted because of (**b**): fracture, (**c**) root resorption, (**d**) caries.

**Figure 2:**
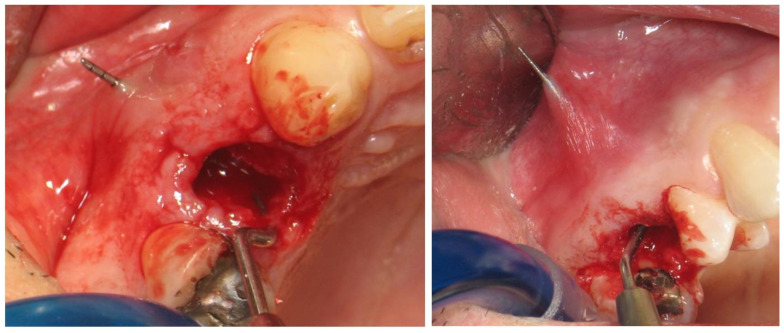
In some cases, bone dehiscence was noticed in the buccal plate and the palatal plate.

**Figure 3:**
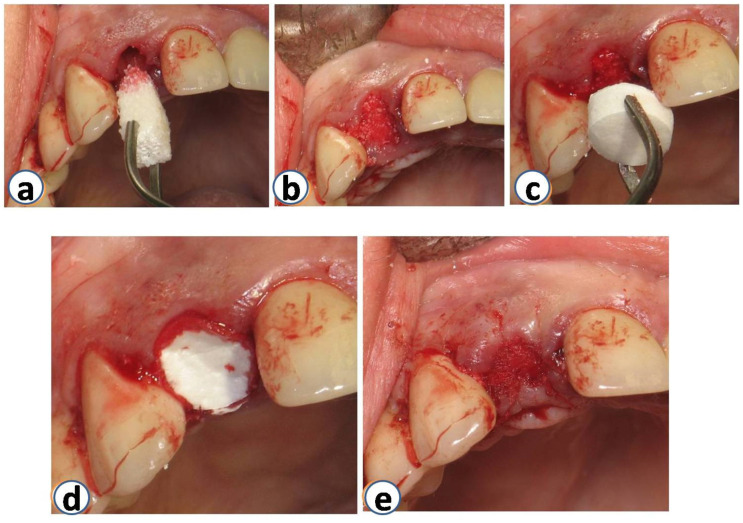
Sites were grafted using DBBM with collagen (Bio-Oss Collagen; Geistlich, Wolhusen, Switzerland) immediately after the extraction (**a**,**b**). Immediately after that, the same sites were covered with a resorbable collagen matrix (Mucograft^®^ Seal; Geistlich, Wolhusen, Switzerland, DMPS Biomaterials) (**c**–**e**).

All surgeries were performed at the Department of Periodontology of Aristotle University of Thessaloniki. Prior to extraction, periapical X-rays and CBCT were performed. On the day of surgery, teeth were extracted non-traumatically, with no buccal flaps raised (Figure 1). In some cases, the buccal plate was missing or had some kind of deficiency (the periodontal probe could penetrate through and through after extraction) (Figure 2). After extraction, the sites were grafted using DBBM combined with collagen (Bio-Oss collagen; Geistlich, Wolhusen, Switzerland) (Figure 3a,b). The site was sutured to keep the graft steady. All sites were then covered with a resorbable collagen matrix (Mucograft^®^ Seal; Geistlich, Wolhusen, Switzerland, DMPS Biomaterials) (Figure 3c,d).

**Figure 4:**
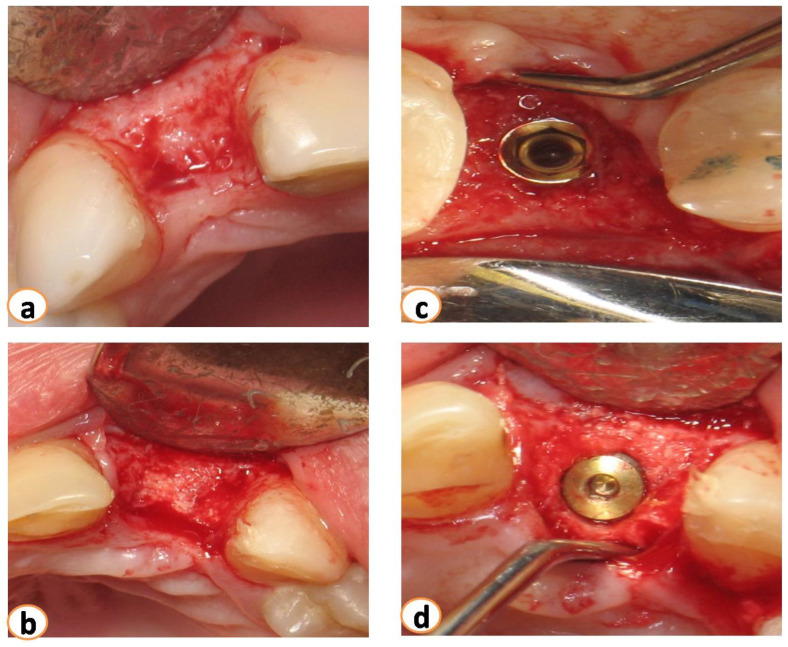
Five months after grafting (**a**). Flaps were raised, (**b**) the area of extraction was filled with new bone, and implants were placed (**c**,**d**).

Approximately five months after grafting, full-thickness flaps were raised, and dental implants were placed adhering to respective company protocols (3i Biomet-Florida-USA, Miami, FL, USA) (Figure 4). In all cases, the diameter of the placed implants ranged from 3.8 to 4.2 mm. All implants were successfully installed, and primary stability was established. Four months after placement, healing abutments were placed. The implants were restored with single-unit screw-retained crowns by the Department of Prosthodontics at the Aristotle University of Thessaloniki. After the final reconstructions, patients were enrolled in the maintenance phase.

### 2.3. Infection and Pain Control

Before surgery, patients were prescribed 2 g of amoxicillin–clavulanic acid and advised to take antibiotics one day before surgery and to continue for six days postoperatively (amoxicillin–clavulanic, 625 mg TID). For pain control, Ibuprofen 600 mg, TID, was also prescribed.

### 2.4. Long-Term Results of Soft Tissue Esthetic Outcome and Radiographic Evaluation

**Figure 5:**
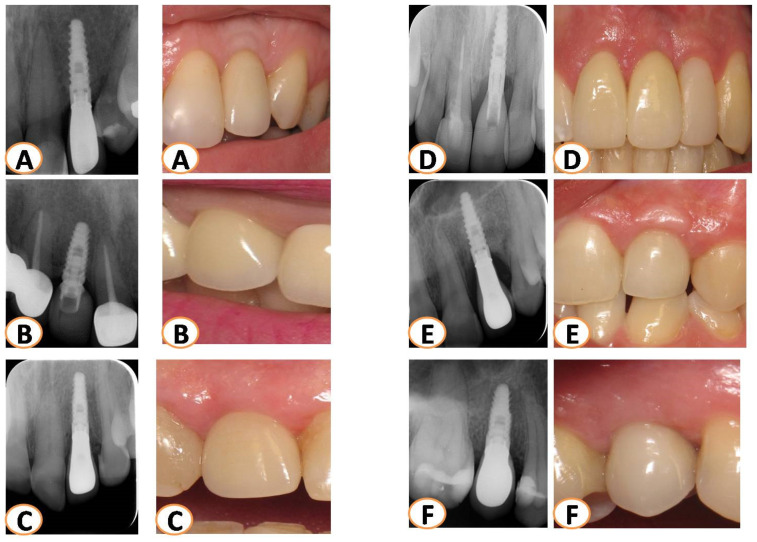
All patients were monitored for more than seven years with X-rays (parallel technique) and pictures. The last X-rays and photos taken were evaluated for each case. Several cases are presented in this figure (**A**–**F**).

**Figure 6:**
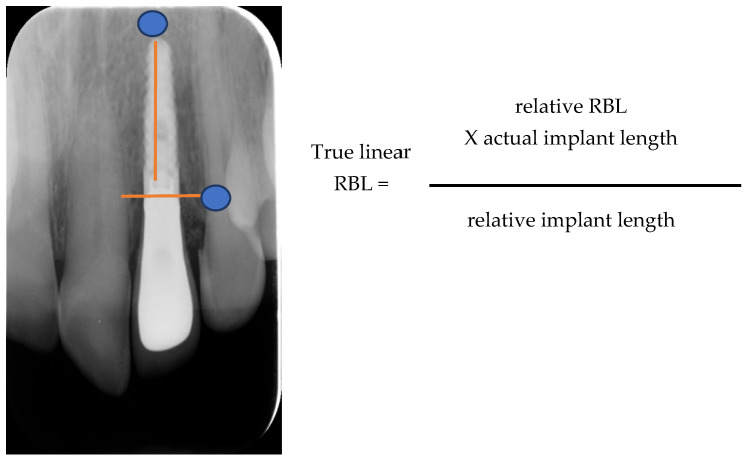
Measuring True Linear Bone Loss.

All patients were monitored via radiographs (parallel technique, probing depths, bone loss measurement, and photographs taken for at least 7 years (Figure 5) [22]. The bone loss (BL) of the implants placed was calculated from the last radiograph taken, as follows: True linear BL = relative BL that was calculated radiographically from the top of the implant, multiplied by the actual implant length, and then divided by the relative radiographic implant length (Figure 6).

**Figure 7:**
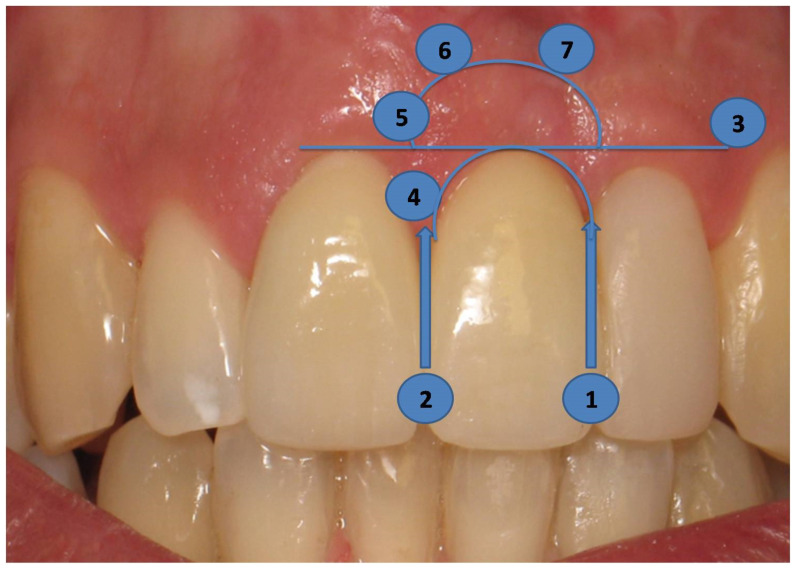
The PES technique uses the pictures taken. It consists of seven variables; the evaluated variables are the mesial (**1**) and distal (**2**) papillae, the level of soft tissue margin (**3**), the soft tissue contour (**4**), the alveolar process (**5**), the soft tissue color (**6**), and the soft tissue texture (**7**).

In order to evaluate the long-term esthetic outcome, the Pink Esthetic Score (PES) technique was employed (Figure 7). Intraoral photographs, used in the PES technique, were taken and evaluated. There are seven variables involved; each ranging between 0 and 2. As a result, the lowest possible score is 0, and the highest can be 14, reflecting a perfect match of the peri-implant soft tissue with the reference tooth. The evaluated variables include the mesial and distal papilla, the level of soft tissue margin, the soft tissue contour, the alveolar process, the soft tissue color, and the soft tissue texture (Figure 7).

The mesial and distal papillae were evaluated for completeness, incompleteness, or absence. All other variables were assessed by comparison with a reference tooth, i.e., the corresponding tooth (anterior region) or a neighboring tooth (premolar region) (Figure 6). Calibration sessions were performed, assessing five cases within 15 days apart, by three independent researchers/periodontal residents of the Department of Periodontology at Aristotle University of Thessaloniki Dental School. The results yielded high reproducibility rates for all three examiners for all variables under consideration (Table 2).

### 2.5. Statistical Analysis

Continuous variables were represented with mean and standard deviation, whereas frequencies and percentages were used for categorical variables. The assumption of normal distribution was investigated for all the variables using the Shapiro–Wilk test. Thus, both parametric and non-parametric tests were used. The Wilcoxon signed-rank test for paired samples was used to compare the mean values of the PES score parameters between the two assessments. The Spearman’s r correlation factor was used to calculate the intra and inter-examiner reproducibility rates. Statistical analysis was performed using STATA 13 (StataCorp LP, College Station, TX, USA). The statistical significance level was set at *p*-value ≤ 0.05.

## 3. Results

The sample in this case series study comprised 15 patients. Three of the 15 patients had buccal deficiency while one patient had a palatal. Nine of the fifteen teeth were extracted because of fracture, two because of external or internal resorption, and the rest because of extended caries. The mean follow-up time was 8.7 years.

Clinically, it was also noticed, at the time of implant placement, that the areas of grafted sites were filled with new bone. All 15 implants placed presented primary stability. After surgery, all patients experienced some swelling. Only three of the 15 cases received provisional removable prosthetic restorations; the rest were provided with a Maryland as a temporary restoration. Four months later, a healing abutment was placed, and implants were prosthetically reconstructed. Adequate keratinized tissue was noticed around all implants (3 to 4 mm) and tissue thickness was more than 4 mm in all cases.

### 3.1. Radiographic Bone Loss

After the final reconstructions, all patients followed the maintenance face. Implants were radiographically monitored (every year) for more than 7 years (mean 8.2 years) (Table 1; Figure 5). A 100% survival rate for all 15 implants was observed. The marginal bone loss that was noticed was minimal (0–0.92 mm) with a mean percentage of 2.25%.

### 3.2. PES Assessment

In each of the two assessments, three observers rated 15 cases, i.e., a total of 45 PESs/assessments. Each examiner performed the assessment twice. The mean PES was 11.40 (±1.44) in the first assessment and 11.38 (±1.63) in the second. The difference was not statistically significant (*p* = 978) [23].

The variables receiving the higher score in both assessments were the alveolar process followed by the soft tissue contour. The differences in the mean values for each independent variable in each assessment did not yield statistical significance (Table 3). Both assessments displayed high reproducibility rates among examiners (Table 4).

## 4. Discussion

After tooth extraction, the alveolar bone structure undergoes significant alterations, accompanied by volumetric changes in the soft tissue. Numerous studies have elucidated these changes, shedding light on the dynamics of post-extraction healing.

A histological study by Araújo et al. delineated the dimensional alterations of the alveolar ridge post-extraction, identifying two distinct phases. Initially, resorption of the bundle bone within the socket occurs, followed by subsequent resorption on the outer surface of the socket wall. These phases collectively contribute to a considerable vertical reduction of the buccal wall [4].

Similarly, Cardaropoli et al. observed histological changes during the healing phase of extraction sockets. Within the first three days, a blood clot predominantly occupies the extraction site, which is gradually replaced by a provisional matrix (PCT) by day seven. By day 14, the socket tissue comprises provisional matrix (PM) and woven bone, transitioning to mineralized bone occupying 88% of the socket volume by day 30. However, this bone tissue decreases to 15% by day 180, with bone marrow (BM) occupying around 75% on day 60, increasing to 85% by day 180 [19].

These histological findings are consistent with clinical studies on humans, demonstrating significant mean changes in the alveolar ridge, both horizontally (approximately 4 mm) and vertically (almost 1 mm), following tooth extraction [18]. Notably, even with atraumatic extraction techniques, such as flapless approaches, long-term follow-ups have not shown benefits in preventing alveolar ridge resorption [4].

In the literature, there has been a proposition that placing implants in fresh extraction sockets could mitigate the hard and soft tissue changes post-tooth loss [20]. However, conflicting evidence exists, with some studies unable to validate this hypothesis [2,4]. This highlights the complexity of post-extraction healing and the need for further research to elucidate the efficacy of implant placement in preserving alveolar ridge dimensions. In order to overcome these adverse results on the alveolar ridge after tooth extraction, a variety of bone grafting materials have been proposed for socket grafting (ARP-SG), including allografts, xenografts, alloplasts, autogenous bone, and various growth factors. Most systematic reviews available agree in favor of strong evidence that ARP-SG reduces alveolar bone resorption in both horizontal and vertical dimensions [6,12,26,29].

A recent systematic review comparing the use of xenograft versus extraction-derived a significant MD of −1.18 mm, with a 95% confidence interval (CI) of −1.82 to −0.54; *p* = 0.0003, I^2^ = 82% in the bucco-lingual/palatal width, and a significant MD of −1.35 mm, with a 95% CI −2.00 to −0.70; *p* < 0.0001, I^2^ = 87% concerning the alveolar ridge height [24].

In the current study, the PE score was used to evaluate the long-term esthetic effect of bone augmentation, with xenograft and collagen matrix in the anterior maxilla. The evaluation yielded a PES of 11.4 in both assessments, indicating a highly esthetic outcome. This is in accordance with previous studies. More specifically, Juodzvalys and Wang derived a PES of 11, one year after immediate implant placement and socket augmentation [25]. Similarly, Chen and co-workers found a PES of 11.1, two years after delayed implant placement in non-augmented bone. However, these are short-term studies [23].Cosyn and co-workers, using immediate implant placement with minimally induced bone augmentation, reported a small amount of recession after three years [30]. To our knowledge, long-term studies on the topic (>5 years) are scarce. In our study, high esthetic outcomes were evident seven years after implant placement, validating the stability of the technique. In addition, the mean PES for both assessments was 11.4, indicating a good esthetic result. As mentioned earlier, esthetic scores of 0–9 represent suboptimal esthetic results, while scores of 10–12 and 13–14 indicate good and optimum esthetics, respectively.

Evaluating implant esthetics with PES is considered an accurate and highly reproducible technique [27,28]. In the current study, the reproducibility of the technique was evaluated using three different calibrated examiners at two separate points in time. In most cases, an ICC > 0.8 was reported, indicating strong agreement among observers. Previous studies have indicated a specialty effect on the esthetic outcomes, with different specialists yielding variable results [27]. This was not the case for the current study, where all observers were trained periodontists with similar clinical experience.

The findings of the present study suggest that grafting the socket of an extracted tooth with Deproteinized Bovine Bone Mineral (DBBM) with collagen, which was then covered by a resorbable collagen matrix (CM), seems to produce predictable and reproducible clinical and radiographic outcomes. The results showed a 100% survival rate for the implants and almost no bone loss for more than seven years, even in cases where the buccal plate was missing. The findings of this study agree with previous studies, indicating that the use of Deproteinized Bovine Bone Mineral (DBBM) with collagen, such as Bio-Oss^®^ Collagen, can preserve the alveolar ridge after extraction [31,32]. Also, the use of collagen matrix (CM) in all cases can improve the healing and regeneration of soft tissues, considering that adequate keratinized tissue (3 to 4 mm) and thick tissues (4 to 5 mm) were noticed in all cases [33,34,35]. This fact is very important since it has been shown that the vertical thickness of soft tissue plays a major part in the etiology of early crestal bone loss, and thick, soft tissue can maintain bone around dental implants with minimal remodeling [36,37,38].

The singularity of this study, which makes it important to clinicians, has been the monitoring for more than seven years and the evaluation of the long-term esthetic, clinical, and radiographic results of this socket preservation technique. Finally, it must be mentioned that no contrast with other grafting materials and techniques was performed. The use of CBCT is a great tool to radiographically evaluate the new bone formation and potential bone loss. In this study, several of the patients did not have a CBCT done to follow up on bone loss, especially those who had their implants placed more than six years ago. Since this study is a retrospective case series, it was decided to use periapical X-rays and clinical measurements to evaluate potential bone loss, bearing in mind that changes in the buccal or palatal bone may not have been detected. Probably, future clinical trials using different grafting materials and combinations are needed to validate the findings of the present study.

## 5. Conclusions

In conclusion, this study shows that when Deproteinized Bovine Bone Mineral (DBBM) was used with collagen, covered by a resorbable collagen matrix (CM), the alveolar ridge was preserved, the implants placed had a 100% survival rate, and the esthetic results were stable in the long-term with minimal radiographic evidence of bone loss. 

## Figures and Tables

**Table 1:** Patients included in the study.

Patients	Tooth	R.F.E	B.D.	Implant Type	W/L m.m.	Years Follow Up	T.L.B.L	% B.L
Patient 1	21	Fracture	NO	3i Biomet	4/13	10	0.92 mm	7.7%
Patient 2	24	Fracture	NO	3i Biomet	4/13	10	0.0 mm	0%
Patient 3	12	Carries	NO	3i Biomet	4/12	10	0.37 mm	2.8%
Patient 4	12	Fracture	NO	Biohorizon	3.8/12	9	0.0 mm	0%
Patient 5	13	Fracture	NO	Biohorizon	3.8/12	7	0.0 mm	0%
Patient 6	22	Carries	NO	Biohorizon	3.0/12	8	0.46 mm	3.8%
Patient 7	21	Fracture	NO	Biohorizon	3.8/12	8	0.46 mm	3.8%
Patient 8	14	Fracture	Buccal	Biohorizon	4.2/12	7	0 mm	0%
Patient 9	22	Fracture	NO	Biohorizon	3.8/12	8	0.55 mm	4.6%
Patient 10	12	Carries	NO	Biohorizon	3.8/12	8	0 mm	0%
Patient 11	22	Resorption	Palatal	Biohorizon	3.8/12	7	0 mm	0%
Patient 12	22	Resorption	NO	Biohorizon	3.8/12	7	0.46 mm	3.8%
Patient 13	15	Fracture	Buccal	Biohorizon	3.8/12	8	0.6 mm	5%
Patient 14	11	Carries	NO	Biohorizon	3.8/12	8	0 mm	0%
Patient 15	15	Fracture	Buccal	Biohorizon	4.2/12	7	0 mm	0%

R.F.E—Reason for extraction; B.D.—bone dehiscence; T.L.B.L—true linear bone loss.

**Table 2:** Intra-examiner reproducibility in each examiner.

1st Assesment			2nd Assesment			
	Examiner 1		Examiner 2		Examiner 3	
Variable	ICC * (95% CI)	*p* Value	ICC (95% CI)	*p* Value	ICC (95% CI)	*p* Value
Mesial Papilla	0.576 (0.088–0.841)	0.12	0.847 (0.690–0.948)	<0.01	1.000	<0.01
Distal Papilla	0.896 (0.708–0.965)	<0.01	0.732 (0.350–0.905)	<0.01	0.930 (0.796–0.977)	<0.01
Level of the soft tissue margin	0.727 (0.345–0.904)	<0.01	0.860 (0.621–0.953)	<0.01	1.000	<0.01
Soft tissue contour	0.772 (0.428–0.921)	<0.01	1.000	<0.01	1.000	<0.01
Color	0.866 (0.634–0.955)	<0.01	0.847 (0.546–0.948)	<0.01	0.896 (0.708–0.965)	<0.01
Texture	0.778 (0.441–0.923)	<0.01	0.930 (0.796–0.977)	<0.01	0.839 (0.727–0.972)	<0.01
Overall	0.732 (0.431–0.905)	<0.01	0.920 (0.804–0.985)	<0.01	0.958 (0.975–0.986)	<0.01

* ICC: Intraclass Correlation Coefficient.

**Table 3:** Mean values of each variable in PES and the differences among subsequent examinations.

	Mean Value (±SD)		
PES Variable	1st Assesment	2nd Assesment	*p* Value
Mesial Papilla	1.69 (±0.46)	1.71 (±0.45)	0.564
Distal Papilla	1.40 (±0.44)	1.42 (±0.63)	0.655
Level of the soft tissue margin	1.64 (±0.48)	1.61 (±0.49)	0.564
Soft tissue contour	1.83 (±0.37)	1.85 (±0.35)	0.317
Alveolar Process	1.95 (±0.21)	1.99 (±0.18)	0.157
Color	1.57 (±0.5)	1.59 (±0.54)	0.554
Texture	1.26 (±0.07)	1.06 (±0.7)	0.981

**Table 4:** Reproducibility among two assessments.

**1st Evaluation**		
**Variable**	ICC * (95% CI)	***p* Value**
Mesial Papilla	0.925 (0.817–0.974)	<0.01
Distal Papilla	0.924 (0.815–0.974)	<0.01
Level of the soft tissue margin	0.879 (0.702–0.958)	<0.01
Soft tissue contour	0.940 (0.853–0.979)	<0.01
Alveolar Process		
Color	0.834 (0.594–0.94)	<0.01
Texture	0.944 (0.843–0.984)	<0.01
Overall	0.935 (0.842–0.977)	<0.01
**2nd Evaluation**		
**Variable**	**ICC (95% CI)**	***p* Value**
Mesial Papilla	0.918 (0.758–0.971)	<0.01
Distal Papilla	0.802 (0.515–0.931)	<0.01
Level of the soft tissue margin	0.877 (0.697–0.977)	<0.01
Soft tissue contour	1.000	<0.01
Color	0.829 (0.581–0.941)	<0.01
Texture	0.969 (0.924–0.989)	<0.01
Overall	0.920 (0.844–0.935)	<0.01

* ICC: Intraclass Correlation Coefficient.

## Data Availability

Data are contained within the article.

## References

[1] Araújo M.G., Silva C.O., Misawa M., Sukekava F. (2015). Alveolar socket healing: What can we learn?. Periodontology 2000.

[2] Botticelli D., Berglundh T., Lindhe J. (2004). Hard-tissue alterations following immediate implant placement in extraction sites. J. Clin. Periodontol..

[3] Farmer M., Darby I. (2014). Ridge dimensional changes following single-tooth extraction in the aesthetic zone. Clin. Oral Implant. Res..

[4] Araujo M.G., Sukekava F., Wennstrom J.L., Lindhe J. (2005). Ridge alterations following implant placement in fresh extraction sockets: An experimental study in the dog. J. Clin. Periodontol..

[5] Thoma D.S., Benić G.I., Zwahlen M., Hämmerle C.H.F., Jung R.E. (2009). A systematic review assessing soft tissue augmentation techniques. Clin. Oral Implant. Res..

[6] MacBeth N., Trullenque-Eriksson A., Donos N., Mardas N. (2017). Hard and soft tissue changes following alveolar ridge preservation: A systematic review. Clin. Oral Implant. Res..

[7] Araújo M.G., Sukekava F., Wennström J.L., Lindhe J. (2006). Tissue modeling following implant placement in fresh extraction sockets. Clin. Oral Implant. Res..

[8] Ferrus J., Cecchinato D., Pjetursson E.B., Lang N.P., Sanz M., Lindhe J. (2010). Factors influencing ridge alterations following immediate implant placement into extraction sockets. Clin. Oral Implant. Res..

[9] Matarasso S., Salvi G.E., Siciliano V.I., Cafiero C., Blasi A., Lang N.P. (2009). Dimensional ridge alterations following immediate implant placement in molar extraction sites: A six-month prospective cohort study with surgical re-entry. Clin. Oral Implant. Res..

[10] Sanz M., Cecchinato D., Ferrús J., Pjetursson E.B., Lang N.P., Lindhe J. (2010). A prospective, randomized-controlled clinical trial to evaluate bone preservation using implants with different geometry placed into extraction sockets in the maxilla. Clin. Oral Implant. Res..

[11] Iocca O., Farcomeni A., Lopez S.P., Talib H.S. (2017). Alveolar ridge preservation after tooth extraction: A Bayesian Network meta-analysis of grafting materials efficacy on prevention of bone height and width reduction. J. Clin. Periodontol..

[12] Majzoub J., Ravidà A., Starch-Jensen T., Tattan M., Del Amo F.S.-L. (2019). The Influence of Different Grafting Materials on Alveolar Ridge Preservation: A Systematic Review. J. Oral Maxillofac. Res..

[13] De Angelis P., De Rosa G., Manicone P.F., De Giorgi A., Cavalcanti C., Speranza A., Grassi R., D’addona A. (2022). Hard and soft tissue evaluation of alveolar ridge preservation compared to spontaneous healing: A retrospective clinical and volumetric analysis. Int. J. Implant. Dent..

[14] Ucer C., Khan R.S. (2023). Extraction Socket Augmentation with Autologous Platelet-Rich Fibrin (PRF): The Rationale for Socket Augmentation. Dent. J..

[15] Araújo M.G., Liljenberg B., Lindhe J. (2010). Dynamics of Bio-Oss^®^ Collagen incorporation in fresh extraction wounds: An experimental study in the dog. Clin. Oral Implant. Res..

[16] Sbordone C., Toti P., Martuscelli R., Guidetti F., Ramaglia L., Sbordone L. (2016). Retrospective volume analysis of bone remodeling after tooth extraction with and without deproteinized bovine bone mineral insertion. Clin. Oral Implant. Res..

[17] Sivolella S., Botticelli D., Prasad S., Ricci S., Bressan E., Prasad H. (2020). Evaluation and comparison of histologic changes and implant survival in extraction sites immediately grafted with two different xenografts: A randomized clinical pilot study. Clin. Oral Implant. Res..

[18] Carmagnola D., Adriaens P., Berglundh T. (2003). Healing of human extraction sockets filled with Bio-Oss^®^. Clin. Oral Implant. Res..

[19] Cardaropoli D., Tamagnone L., Roffredo A., Gaveglio L., Cardaropoli G. (2012). Socket preservation using bovine bone mineral and collagen membrane: A randomized controlled clinical trial with histologic analysis. Int. J. Periodontics Restor. Dent..

[20] Jung R.E., Hürzeler M.B., Thoma D.S., Khraisat A., Hämmerle C.H.F. (2011). Local tolerance and efficiency of two prototype collagen matrices to increase the width of keratinized tissue. J. Clin. Periodontol..

[21] Thoma D.S., Villar C.C., Cochran D.L., Hämmerle C.H.F., Jung R.E. (2012). Tissue integration of collagen-based matrices: An experimental study in mice. Clin. Oral Implant. Res..

[22] Thoma D.S., Gasser T.J.W., Hämmerle C.H.F., Strauss F.J., Jung R.E. (2023). Soft tissue augmentation with a volume-stable collagen matrix or an autogenous connective tissue graft at implant sites: Five-year results of a randomized controlled trial post implant loading. J. Periodontol..

[23] Chen S.T., Buser D. (2009). Clinical and esthetic outcomes of implants placed in postextraction sites. Int. J. Oral Maxillofac. Implant..

[24] A Atieh M., Alsabeeha N.H., Payne A.G., Duncan W., Faggion C.M., Esposito M. (2015). Interventions for replacing missing teeth: Alveolar ridge preservation techniques for dental implant site development. Emergencias.

[25] Juodzbalys G., Wang H. (2007). Soft and hard tissue assessment of immediate implant placement: A case series. Clin. Oral Implant. Res..

[26] Darby I., Chen S.T., Buser D. (2009). Ridge preservation techniques for implant therapy. Int. J. Oral Maxillofac. Implant..

[27] Gehrke P., Lobert M., Dhom G. (2008). Reproducibility of the pink esthetic score—Rating soft tissue esthetics around single-implant restorations with regard to dental observer specialization. J. Esthet. Restor. Dent..

[28] Cho H.-L., Lee J.-K., Um H.-S., Chang B.-S. (2010). Esthetic evaluation of maxillary single-tooth implants in the esthetic zone. J. Periodontal Implant. Sci..

[29] Canullo L., Pesce P., Antonacci D., Ravidà A., Galli M., Khijmatgar S., Tommasato G., Sculean A., Del Fabbro M. (2022). Soft tissue dimensional changes after alveolar ridge preservation using different sealing materials: A systematic review and network meta-analysis. Clin. Oral Investig..

[30] Cosyn J., De Lat L., Seyssens L., Doornewaard R., Deschepper E., Vervaeke S. (2019). The effectiveness of immediate implant placement for single tooth replacement compared to delayed implant placement: A systematic review and meta-analysis. J. Clin. Periodontol..

[31] Iorio-Siciliano V., Ramaglia L., Blasi A., Bucci P., Nuzzolo P., Riccitiello F., Nicolò M. (2020). Dimensional changes following alveolar ridge preservation in the posterior area using bovine-derived xenografts and collagen membrane compared to spontaneous healing: A 6-month randomized controlled clinical trial. Clin. Oral Investig..

[32] Morelli T., Zhang S., Monaghan E., Moss K., Lopez B., Marchesan J. (2020). Three-Dimensional Volumetric Changes After Socket Augmentation with Deproteinized Bovine Bone and Collagen Matrix. Int. J. Oral Maxillofac. Implant..

[33] Tavelli L., McGuire M.K., Zucchelli G., Rasperini G., Feinberg S.E., Wang H., Giannobile W.V. (2020). Extracellular matrix-based scaffolding technologies for periodontal and peri-implant soft tissue regeneration. J. Periodontol..

[34] Rocchietta I., Schupbach P., Ghezzi C., Maschera E., Simion M. (2012). Soft tissue integration of a porcine collagen membrane: An experimental study in pigs. Int. J. Periodontics Restor. Dent..

[35] Schlee M., Ghanaati S., Willershausen I., Stimmlmayr M., Sculean A., Sader R.A. (2012). Bovine pericardium based non-cross linked collagen matrix for successful root coverage, a clinical study in human. Head Face Med..

[36] Linkevicius T., Puisys A., Steigmann M., Vindasiute E., Linkeviciene L. (2015). Influence of Vertical Soft Tissue Thickness on Crestal Bone Changes Around Implants with Platform Switching: A Comparative Clinical Study. Clin. Implant. Dent. Relat. Res..

[37] Del Amo F.S., Lin G., Monje A., Galindo-Moreno P., Wang H. (2016). Influence of Soft Tissue Thickness on Peri-Implant Marginal Bone Loss: A Systematic Review and Meta-Analysis. J. Periodontol..

[38] Thoma D.S., Naenni N., Figuero E., Hämmerle C.H.F., Schwarz F., Jung R.E., Sanz-Sánchez I. (2018). Effects of soft tissue augmentation procedures on peri-implant health or disease: A systematic review and meta-analysis. Clin. Oral Implant. Res..

